# Supplementary material for: Whole-Genome Sequencing of a Potentially Novel Aeromonas Species Isolated from Diseased Siberian Sturgeon (Acipenser baerii) Using Oxford Nanopore Sequencing
Source: Microorganisms. 2025 Jul 17;13(7):1680. doi: 10.3390/microorganisms13071680 (PMC12300149; doi:10.3390/microorganisms13071680)
Supplement: Supplementary file 1 [file microorganisms-13-01680-s001.zip › microorganisms-3653881-supplementary.pdf]

SUPPLEMENTARY MATERIAL

Whole Genome Sequencing of a Potentially Novel *Aeromonas* Species Isolated from Diseased Siberian Sturgeon (*Acipenser baerii*) Using Oxford Nanopore Sequencing

Akzhigit Mashzhan, Izat Smekenov, Serik Bakiyev, Kalamkas Utegenova, Diana Samatkyzy, Asset Daniyarov, Ulykbek Kairov, Dos Sarbassov and Amangeldy Bissenbaev

Table S1. Genomes and GenBank accession numbers used for GGDC and OrthoANI analyses

Strain	GenBank/RefSeq assembly accessions
Aeromonas hydrophila subsp. *ranae* CIP 107985	GCA_000820325.1
Aeromonas hydrophila ATCC 7966^T	GCF_000014805.1
***Aeromonas oralensis* AB005^T**	CP187186
Aeromonas dhakensis CECT 7289^T	GCF_000819705.1
Aeromonas dhakensis CIP 107500	GCF_000820305.1
Aeromonas jandaei CECT 4228^T	GCA_000819955.1
Aeromonas piscicola LMG 24783^T	GCA_000820005.1
Aeromonas bestiarum CECT 4227	GCA_000819875.1
Aeromonas salmonicida subsp. *pectinolytica DSM 12609^T*	GCA_000820045.1
Aeromonas caviae CECT 4221	GCA_000820265.1
Aeromonas caviae NCTC 12244	GCA_900476005.1
Aeromonas sanarellii LMG 24682^T	GCA_000820085.1
Aeromonas media CECT 4232	GCA_000819985.1
Aeromonas aquatica AE235^T	GCA_000819695.1
Aeromonas eucrenophila CECT 4224	GCA_000819865.1
Aeromonas encheleia CECT 4342^T	GCA_000819805.1
Aeromonas molluscorum CECT 5864^T	GCA_000819665.1
